# Progressive chorioretinal involvement in a patient with light-chain (AL) amyloidosis: a case report

**DOI:** 10.1186/s12886-020-01341-z

**Published:** 2020-02-21

**Authors:** Edouard Augstburger, José-Alain Sahel, Isabelle Audo

**Affiliations:** 1 Centre Hospitalier National d’Ophtalmologie des Quinze-Vingts, Centre de Maladies Rares “dystrophies rétiniennes d’origine génétique”, DHU Sight Restore INSERM-DHOS CIC 1423, 28, rue de Charenton, 75012 Paris, France; 2 Sorbonne Université, INSERM, CNRS, Institut de la Vision, 17 rue Moreau, F-75012 Paris, France; 3 Department of Ophthalmology, The University of Pittsburgh School of Medicine, Pittsburgh, PA 15213 USA

**Keywords:** AL amyloidosis, Chorioretinal involvement, Drusenoid deposits, Serous retinal detachment, Nephrotic syndrome

## Abstract

**Background:**

To report an unusual case of light-chain (AL) amyloidosis with progressive bilateral chorioretinal abnormalities documented with short-wavelength autofluorescence, SD-OCT, fluorescein and indocyanine green angiography.

**Case presentation:**

Case report of a forty-three-year-old male patient with kappa AL amyloidosis. The patient presented with rapidly progressing pigmented and hyperautofluorescent drusenoid deposits in both eyes, associated with central serous retinal detachments, a pachychoroid and choriocapillaris enlargement. The general assessment revealed a renal failure symptomatic of a nephrotic syndrome, associated with proteinuria composed mainly of free kappa light chains. A kidney biopsy confirmed the diagnosis of kappa AL amyloidosis. Chemotherapy was quickly started. During remission, the extension of drusenoid deposits on the fundus was stopped and a disappearance of the subretinal fluid on SD-OCT was observed.

**Conclusions:**

AL amyloidosis is an insidious and potentially fatal condition. This case is one of the first to document the rapid progression of fundus alterations and their stabilization after disease remission. Identifying these specific fundus abnormalities is essential to avoid diagnosis wandering and therapeutic delay.

## Background

The term amyloidosis gathers an heterogenous group of disorders characterized by the extracellular deposition of amyloid fibrils. Among these, Light-chain (AL) amyloidosis, also known as primary amyloidosis, is the most common form. It is a rare systemic disease with an estimated annual incidence of 9.7 to 14.0 cases per million people [1, 2]. It is characterized by the extracellular tissue deposition of amyloid fibrils derived from kappa or lambda monoclonal light chains. These proteins are synthesized by a clonal population of plasma cells in the bone marrow [3]. Excluding the central nervous system, amyloid deposits can affect and damage all organs and more commonly the heart, kidneys, liver. The disease progresses insidiously, and the general symptoms are usually non-specific (asthenia or dyspnea) often causing diagnostic delay. Unlike secondary (AA) amyloidosis, which does not affect the ocular globe, AL amyloidosis can involved various ocular tissues including the conjunctiva [4], the eyelids [5], the extrinsic ocular muscles [6] or even the temporal artery [7, 8] (manifestations which resemble Horton disease). However, these ophthalmic manifestations are rare and probably underdiagnosed. In a recent review of 68 cases of AL amyloidosis who received a complete ophthalmic examination, Reynold et al. [9] identified eight cases with ocular involvement but only one with specific fundus abnormalities. Here, we report a rare case of AL amyloidosis diagnosed secondary to progressive decreased vision in relation with chorioretinal alterations, as imaged with fundus autofluorescence and Spectral domain-Optical coherence tomography (SD-OCT).

## Case presentation

**Fig. 1:**
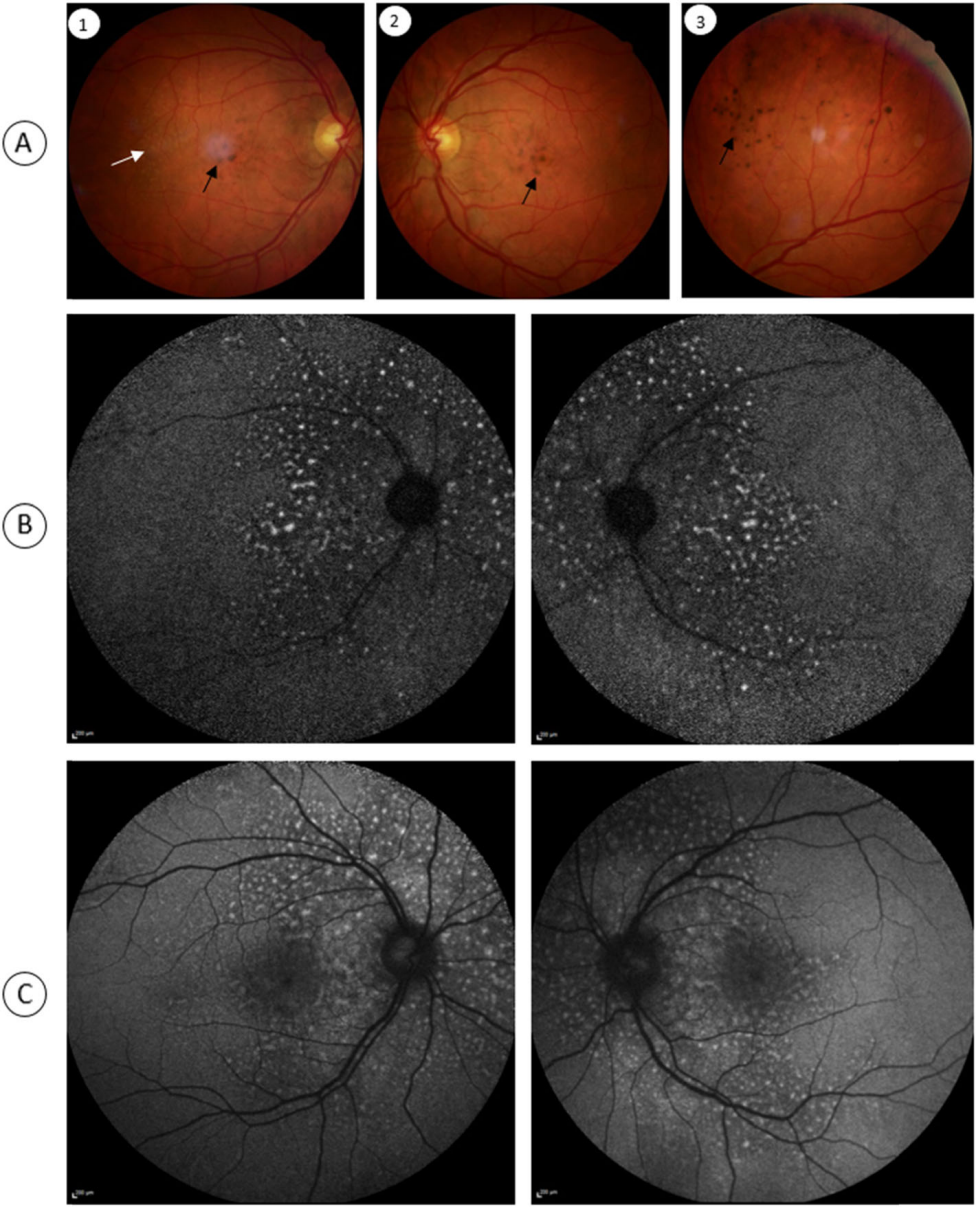
Fundus and autofluorescence features of a patient with chorioretinal lesions due to primary amyloid light-chain amyloidosis. Pigmented (black arrows) and yellowish unpigmented (white arrow) spots are visible at the posterior pole of the right (A1) and left (A2) eyes. These lesions are also found in the mid periphery (A3). Near infrared (B) and short wavelength (C) fundus autofluorescence imaging reveal multiple hyperautofluorescent spots over the posterior pole in both eyes

**Fig. 2:**
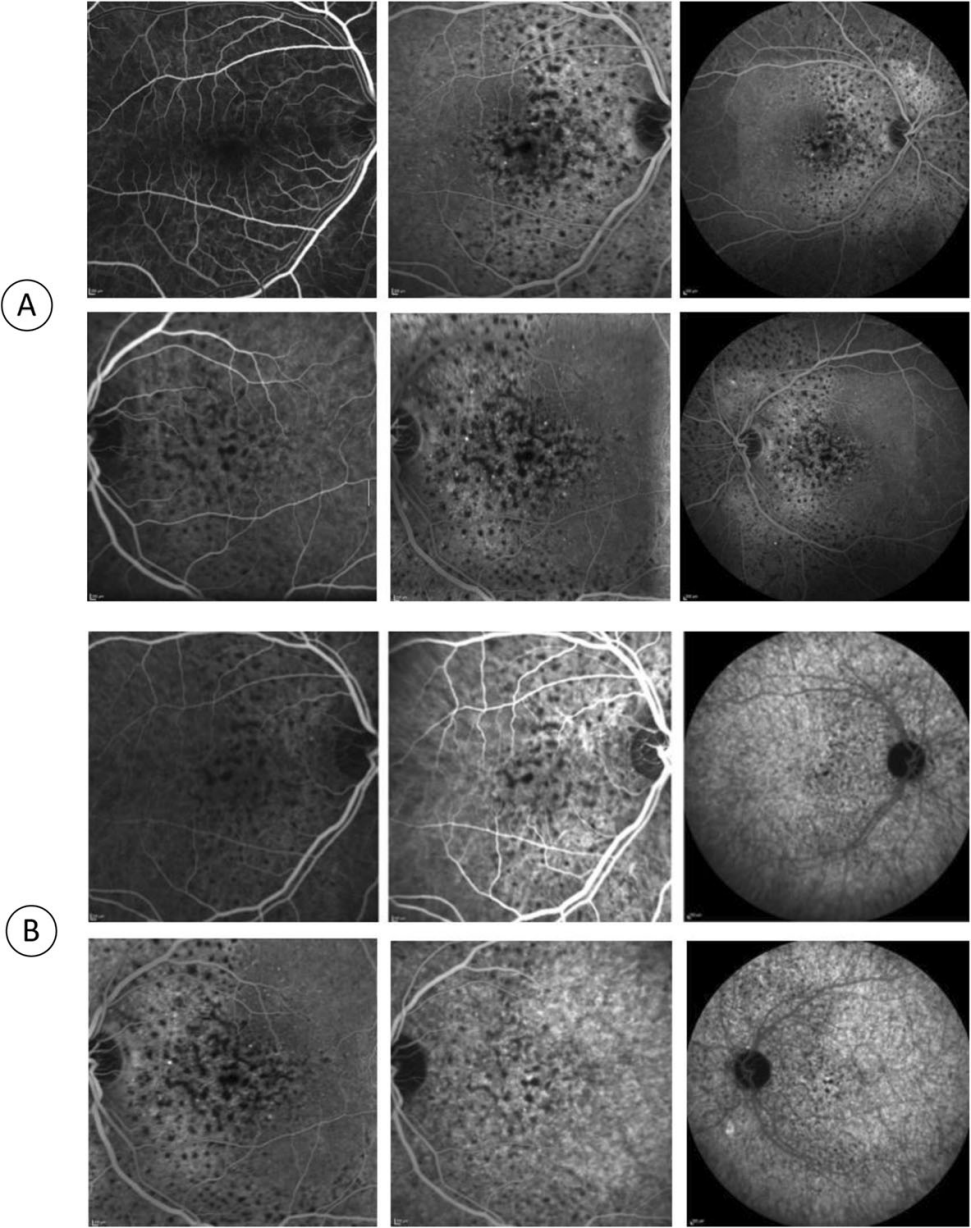
Angiographic features. In fluorescein (**a**) and indocyanine green (**b**) angiography, a masking effect of the material can be observed from the early stages, without diffusion or choriocapillary ischemia

**Fig. 3:**
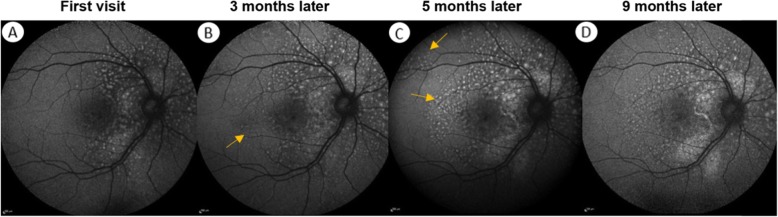
Evolution of the damage to the right eye monitored by short wave length fundus autofluorescence. **a** Multiple hyper-autofluorescent lesions with a peripapillary distribution are present. **b** At diagnosis, 3 months later, extension of the lesions at the posterior pole (yellow arrows) can be observed. This phenomenon continues during the first chemotherapy treatment with an area of increased autofluorescence around the optic nerve **c**. At the time of remission (9 months later), progression of the lesions had stopped, but there is a persistence of the hyperautofluorescent spots

A 43-year-old male was referred to the medical retina clinic with a presumed diagnosis of pattern dystrophy. He had experienced a progressive visual acuity loss that had been worsening over 2 to 3 years, associated with weight loss, dyspnea and fatigue. He had no relevant medical history despite a medically controlled hypercholesterolemia. Ocular mobility was normal and the patient had no complain of diplopia. Best-corrected visual acuity was 20/32 for both eyes with − 1 (− 1.5)75° for the right and − 0.5 (− 2.50) 90° for the left eye. Visual field testing was within normal limit. There were neither eyelid abnormalities nor conjunctival changes. Anterior segments were unremarkable and the intraocular pressure was normal. Fundus examination however revealed well-defined, pigmented clumps at the macula and yellowish spots in the mid-periphery which were better seen along the superior vascular arcades in both eyes (Fig. 1). Near infrared and short wavelength fundus autofluorescence imaging revealed multiple hyperautofluorescent spots over the posterior pole expanding nasal to the optic disc in both eyes On fluorescein and indocyanine green angiography, these lesions were hypofluorescent with a masking effect. There was no evidence of diffusion or choriocapillary ischemia (Fig. 2). Before the diagnosis (after 3 months follow-up), the hyperautofluorescent spots were more numerous in both eyes (Fig. 3).

**Fig. 4:**
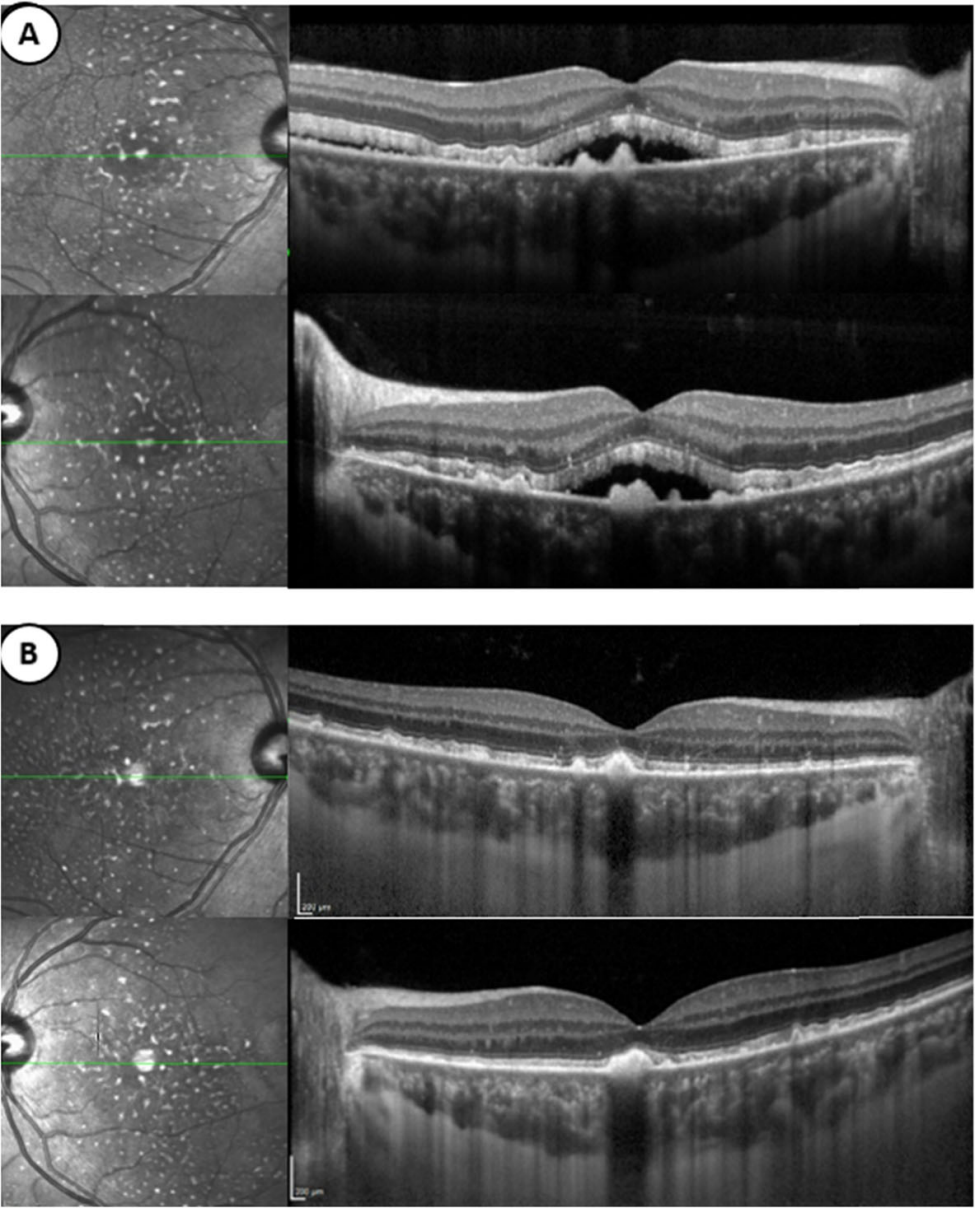
Infrared reflectance image and SD-OCT horizontal scan passing through the fovea of the right and left eyes. **a** At the first visit, the presence of subretinal fluid associated with hyper-reflective deposits in front of the pigment epithelium can be observed. There is also a pachychoroid associated with enlargement of the choriocapillaris. **b** After eight cycles of chemotherapy (9 months from the first visit), the patient is considered to be in remission. Subretinal fluid have disappeared. However, the subretinal deposits are still present and choroidal thickness is unchanged

The SD-OCT (Spectralis® OCT, Heidelberg Engineering, Dossenheim, Germany) revealed bilateral serous retinal detachments (SRD), in which outer segment structures appear elongated and heterogeneous with the presence of subretinal hyper-reflective deposits. In addition, the choroidal thickness was increased (432 μm) with an enlarged choriocapillaris (52 μm) (Fig. 4).

With this atypical presentation, a systemic work-up was ordered which revealed a moderate hypertension, a nephrotic syndrome with a hypercreatinemia and a proteinuria mainly composed of free kappa light chains. A kidney biopsy with Congo red coloration showed amyloid deposits confirming the diagnosis of kappa AL amyloidosis. The patient was started on chemotherapy which consists of 8 cycles of Velcade (2.2 mg/m^2^), Endoxan (500 mg) and dexamethasone (20 mg).

After these eight cycles the patient was considered to be in complete remission with a normalization of the free kappa and lambda light chains (kappa/lambda ratio of 1.73) in peripheral blood but a persistent mild glomerular proteinuria without nephrotic syndrome and hypercreatininemia. His asthenia and dyspnea had disappeared.

On follow-up visits, BCVA improved to 20/25 in both eyes. After an initial progression of the chorioretinal lesions, the hyperautofluorescent spots remain stable (Fig. 3). Retinal detachments resolved in both eyes with the persistence of hyper-reflective retinal deposits on SD-OCT. The choroidal thickness (429 μm) remained unchanged on EDI, as did the expansion of the choriocapillary.

## Discussion and conclusions

The infiltration of light chains at the systemic level is insidious and can affect most tissues; however, ocular involvement is rarely the mode of discovery of AL amyloidosis, particularly through retinal damage, such as in our case. The incidence of this clinical presentation is probably underestimated as it is largely overlooked in these patients. To our knowledge, among the published cases of AL amyloidosis with chorioretinal disease [10–12], our case is one of the first to document the rapid progression of fundus alterations and their stabilization after disease remission. Elementary lesions in our case include pachychoroid, large subretinal detachments, which resolved after chemotherapy and subretinal deposits that are hyperautofluorescent leading to masking on angiography. These features were distinct from pattern dystrophy and Stargardt disease that usually do not manifest with subretinal fluid. The subretinal deposits observed in this case are different from those found in the membranoproliferative glomerulonephritis [13, 14]. The pachychoroid feature with a widened choriocapillaris was first outlined by Roybal [11] and may be secondary to light chain deposition as suggested in several postmortem histological studies [15, 16]. In particular in the latter study, electron microscopic and immunohistochemistry from ocular specimens of a patient with kappa AL amyloidosis revealed kappa light chain deposits within Bruch membrane and in the innermost part of the choroid associated with choriocapillaris obstructions and macular exudative retinal detachments. These alterations would lead to secondary retinal pigment epithelium (RPE) dysfunction accounting for subretinal detachments and impaired photoreceptor outer segment phagocytosis leading to hyperautofluorescent subretinal material accumulation. The nephrotic syndrome could also have a role in the choroidal changes and subretinal detachments through a modification of the oncotic pressure [12, 17–19]. By restoring the plasmatic protein balance and decreasing amyloid deposits, the initiation of the first chemotherapy cycles led to the healing of the nephrotic syndrome and the resorption of the subretinal fluid. In addition, a better RPE/outer retinal interaction stopped the progression of subretinal deposits.

However, even after 9 months of follow-up, visual acuity did not exceed 20/25 due to persistent alterations at the RPE/ photoreceptor interface.

Of note, no change in choroidal thickness was observed after treatment, suggesting that the pachychoroid is linked to amyloid infiltration and not oncotic pressure changes secondary to the nephrotic syndrome. From a histological point of view, the choroid and renal glomerulus share many characteristics [20], explaining their common involvement in diseases with light chain deposits. Amyloid deposits are known to persist throughout life in kidneys of patients with AL amyloidosis. The same might be true for the choroid. Similarly, amyloid deposits have been found on the retina in diseases such as Alzheimer’s disease and cerebral amyloid angiopathy [21, 22].

This case demonstrates the link between subretinal deposits and active amyloid infiltration. Surveillance of the fundus and fundus autofluorescence imaging could be useful means of assessing disease activity and evaluating the response to treatment. Furthermore, patients with atypical deposits with rapid progression should receive a general check-up and a complete systemic examination to avoid diagnostic delay and management of this insidious and potentially lethal condition.

## Data Availability

All the data supporting our findings is contained within the manuscript.

## Abbreviations

BCVA: Best corrected visual acuity
SD-OCT: Spectral domain optical coherence tomography
SRD: Serous retinal detachment

## References

[1] Quock TP, Yan T, Chang E, Guthrie S, Broder MS (2018). Epidemiology of AL amyloidosis: a real-world study using US claims data. Blood Adv.

[2] Desport E, Bridoux F, Sirac C (2012). Al amyloidosis. Orphanet J Rare Dis.

[3] Rosenzweig M, Landau H (2011). Light chain (AL) amyloidosis: update on diagnosis and management. J Hematol Oncol.

[4] Martel A, Oberic A, Moulin A, Tieulie N, Hamedani M (2018). Clinical, radiological, pathological features, treatment and follow-up of periocular and/or orbital amyloidosis: report of 6 cases and literature review. J Fr Ophtalmol.

[5] Iijima S (1992). Primary systemic amyloidosis: a unique case complaining of diffuse eyelid swelling and conjunctival involvement. J Dermatol.

[6] Shah VS, Cavuoto KM, Capo H, Grace SF, Dubovy SR, Schatz NJ (2016). Systemic amyloidosis and Extraocular muscle deposition. J Neuroophthalmol.

[7] Neri A, Rubino P, Macaluso C, Gandolfi SA (2013). Light-chain amyloidosis mimicking giant cell arteritis in a bilateral anterior ischemic optic neuropathy case. BMC Ophthalmol.

[8] Audemard A, Boutemy J, Galateau-Salle F, Macro M, Bienvenu B (2012). AL amyloidosis with temporal artery involvement simulates giant-cell arteritis. Joint Bone Spine.

[9] Reynolds MM, Veverka KK, Gertz MA (2018). Ocular manifestations of systemic amyloidosis. Retina.

[10] Pece A, Yannuzzi L, Sannace C, Scassellati Sforzolini B, Brancato R (2000). Chorioretinal involvement in primary systemic nonfamilial amyloidosis. Am J Ophthalmol.

[11] Roybal CN, Sanfilippo CJ, Nazari H (2015). Multimodal imaging of the retina and choroid in systemic amyloidosis. Retin Cases Brief Rep.

[12] Izzedine H, Fardeau C, Gauthier M (2014). Bilateral serous retinal detachment as a presenting sign of Nephrotic syndrome. Intern Med.

[13] Kheir V, Dirani A, Halfon M, Venetz J-P, Halabi G, Guex-Crosier Y (2017). Multimodal imaging of retinal pigment epithelial detachments in patients with C3 glomerulopathy: case report and review of the literature. BMC Ophthalmol.

[14] Takei M, Obana A, Inomata T (2018). Fundus changes in type III membranoproliferative glomerulonephritis: a case report. BMC Ophthalmol.

[15] Ts’o MO, Bettman JW (1971). Occlusion of choriocapillaris in primary nonfamilial amyloidosis. Arch Ophthalmol.

[16] Schwartz MF, Green WR, Michels RG, Kincaid MC, Fogle J (1982). An unusual case of ocular involvement in primary systemic nonfamilial amyloidosis. Ophthalmology..

[17] Zhang W, Zhang Y, Kang L, Gu X, Wu H, Yang L. Retinal and choroidal thickness in paediatric patients with hypoalbuminaemia caused by nephrotic syndrome. BMC Ophthalmol. 2019;19. 10.1186/s12886-019-1050-0. 10.1186/s12886-019-1050-0 PMC6364472 30727992

[18] De Benedetto U, Pastore MR, Battaglia Parodi M, Bandello F, Pierro L (2012). Retinal involvement in nephrotic syndrome secondary to minimal change disease. Eur J Ophthalmol.

[19] Bilge AD, Yaylali SA, Yavuz S, Simsek İB (2018). Bilateral serous macular detachment in a patient with nephrotic syndrome. Retin Cases Brief Rep.

[20] D’Souza YB, Short CD (2009). The eye--a window on the kidney. Nephrol Dial Transplant.

[21] den Haan J, Morrema THJ, Verbraak FD (2018). Amyloid-beta and phosphorylated tau in post-mortem Alzheimer’s disease retinas. Acta Neuropathol Commun.

[22] Dumitrascu OM, Okazaki EM, Cobb SH (2018). Amyloid-Beta-related Angiitis with distinctive Neuro-ophthalmologic features. Neuroophthalmology..

